# Steroid Avoidance With Low-Dose Tacrolimus is Safe and Effective in the Long-Term for Kidney Transplant Recipients

**DOI:** 10.1016/j.ekir.2025.06.016

**Published:** 2025-06-19

**Authors:** Jana Ekberg, Anna-Elisabeth Aagaard Enevoldsen, Carin Wallquist, Karin Skov, Bente Jespersen, Per Lindnér, Seema Baid-Agrawal

**Affiliations:** 1 Transplant Institute, Sahlgrenska University Hospital, Institute of Clinical Sciences, Sahlgrenska Academy, University of Gothenburg, Gothenburg, Sweden; 2 Department of Nephrology, Aarhus University Hospital, Aarhus, Denmark; 3 Department of Nephrology, Skåne University Hospital, Malmö, Sweden

**Keywords:** kidney transplantation, long-term follow-up, SAILOR study, steroid avoidance

## Abstract

**Introduction:**

In our previous multicenter, open-label, randomized controlled trial (RCT), the SAILOR study, we reported good feasibility, safety, and efficacy of steroid avoidance (SA) at 2 years in immunologically low-risk kidney transplant recipients. A total of 222 participants were randomized to either antithymocyte globulin (ATG) induction + low-dose tacrolimus + mycophenolate mofetil (MMF) or basiliximab induction + low-dose tacrolimus + MMF + prednisolone. Long-term results are needed to confirm the extended safety and efficacy of the SA protocol beyond the short- to medium-term follow-up seen in current reports using low-dose tacrolimus.

**Methods:**

In the SAILOR follow-up observational study, we collected clinical data of 215 participants of the original SAILOR trial at 1, 2, 5 years, and at the last follow-up.

**Results:**

The mean follow-up time postrandomization was 7.3 years. Death-censored graft survival (91.8 vs. 93.1%, *P* = 0.88), patient survival (88 vs. 93%, *P* = 0.32), cumulative incidence of biopsy-proven rejection (19.8% vs. 16.3%, *P* = 0.6), and kidney function (estimated glomerular filtration rate [eGFR]: 50.8 vs. 54 ml/min per 1.73 m^2^, *P* = 0.27) were similar in the 2 arms. Cumulative incidence of posttransplantation diabetes mellitus in per-protocol population was significantly lower in the steroid-avoidance arm. Serious infections requiring hospitalization, and malignancies did not differ significantly. Two-thirds of participants in the SA arm remained on the steroid-free protocol at the end of follow-up.

**Conclusion:**

SA proved to be safe and effective in patients with low immunological risk for up to 7 years following kidney transplantation. Our findings provide robust evidence supporting SA strategy with low-dose tacrolimus without compromising outcomes even at the extended 7-years follow up.

## Graphical Abstract

**Figure:**
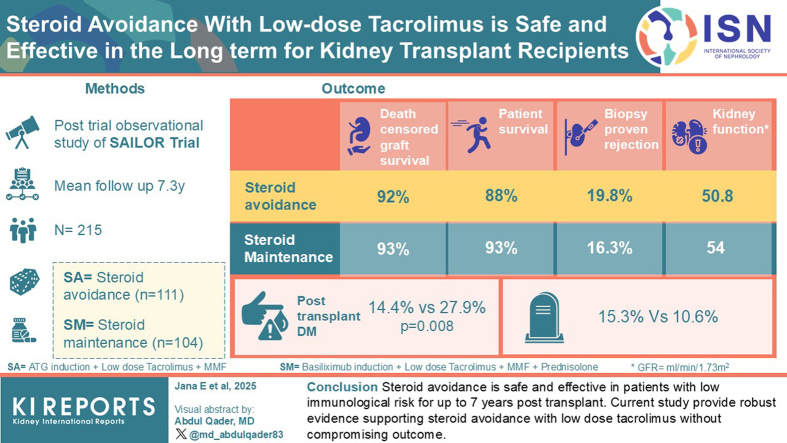

See Commentary on Page 2905

Kidney transplant outcomes have improved with significant advancements in immunosuppressive treatment over the past few decades. At present, the widely used maintenance immunosuppressive regimen after kidney transplantation consists of a calcineurin inhibitor, mainly low-dose tacrolimus; an antimetabolite, mainly MMF; and corticosteroids, preceded by induction with ATG or an interleukin-2 receptor inhibitor and a steroid bolus. This therapy is based on the results of a landmark RCT, the Symphony study.^1^

Because corticosteroids are associated with both short- and long-term adverse effects, including posttransplantation diabetes mellitus (PTDM) as well as cardiovascular morbidity and mortality, various strategies have been implemented to avoid or withdraw steroids, however, with varying results.^2^, ^3^, ^4^, ^5^, ^6^ A generally recognized concern with steroid weaning strategies is a higher risk of acute rejection. A 2016 Cochrane systematic review reached this conclusion based on evidence from 48 RCTs of diverse immunosuppressive regimens—including cyclosporine-based immunosuppression regimens in earlier studies—and only short-term follow-up.^7^

A recent multicenter RCT, the Harmony study, compared rapid steroid withdrawal (SWD) administered in 2 treatment arms using 2 induction agents (ATG and basiliximab) with a control arm, steroid maintenance (SM) with basiliximab induction, in combination with low-dose tacrolimus in all arms. This study found that the 2 rapid SWD regimens were equally effective in preventing biopsy-proven acute rejection at 1-year post-transplant compared with SM-basiliximab treatment.^8^ In line with these findings, our multicenter SAILOR RTC also demonstrated the feasibility, safety, and efficacy of SA extending to 2-years posttransplant in immunologically low-risk kidney recipients using the low-dose tacrolimus + MMF maintenance regimen. The SA protocol with ATG induction was compared with an SM protocol with basiliximab induction. The incidence of PTDM was found to be similarly low in SA and SM arms (12.4% vs. 18.3%, respectively, *P* = 0.3), without an increased risk of overall biopsy-proven rejections (15% vs. 13.8%, respectively, *P* = 0.85).^9^ The composite outcome of freedom from acute rejection, graft loss, and death at 2 years was also similar between the 2 arms.

The only study that has evaluated extended efficacy and safety of SA up to 5 years using low-dose tacrolimus, the current standard-of-care, is Harmony-FU study.^10^ Further studies with even longer follow-up are needed to support the widespread adoption of SA to minimize the long-term side effects associated with steroid use. Therefore, in an observational follow-up investigation, SAILOR FU, we sought to evaluate the impact of SA on long-term efficacy and safety outcomes in patients included in the original SAILOR study after a 7-year follow-up period.

## Methods

### Study Design and Patient Population

The original SAILOR study was an investigator-initiated, multicenter, open-label RCT in immunologically low-risk (panel reactive antibodies < 25%) kidney transplant recipients, with a 2-year follow-up duration, conducted at 3 Scandinavian transplant centers (Gothenburg and Malmö, Sweden; and Aarhus, Denmark) (EudraCT nr. 2012-000451-13). The participants in the SAILOR RCT were randomized in one of the following 2 treatment arms: (i) SA: induction with ATG (2.5 mg/d on days 0 and 1; total 5 mg) and methylprednisolone bolus before ATG (250 mg on day 0 and 50 mg on day 1), and maintenance with tacrolimus and MMF. (ii) SM: induction with basiliximab (20 mg/d on days 0 and 4; total 40 mg) and methylprednisolone bolus 500 mg (day 0), and maintenance based on tacrolimus, MMF, and prednisolone. All patients received *Pneumocystis jirovecii* pneumonia prophylaxis with trimethoprim-sulfamethoxazole, all cytomegalovirus IgG-negative recipients receiving kidney from IgG-positive donors obtained prophylaxis with valganciclovir for 6 months, and IgG-positive recipients in Gothenburg and Malmoe received prophylaxis for 3 months.

All SAILOR RCT participants provided written informed consent containing information about the observational follow-up. The study was approved by the National Ethical Board in Sweden (Dnr. 357-12; 2019-01763; 2022-02860-02) and the Regional Ethical Board in Denmark (Dnr. 1-10-72-211-13). The inclusion and exclusion criteria have been described in our previous paper.^9^

The current observational SAILOR FU study was conducted to compare the long-term safety and efficacy outcomes of SA or SM in kidney transplant recipients who received low-dose tacrolimus and MMF and participated in the original SAILOR RTC.

All participants of the SAILOR RTC were included, except those who withdrew written consent. Kidney transplant recipients were followed-up with beyond the final 2-year SAILOR study visit, according to each center's standard of care.

### Data Collection

Follow-up data were collected between December 11, 2022 and November 10, 2023, which included laboratory data (which had not been collected in the original study) at 1-, 2-, and 5-years posttransplant, as well as at the last recorded clinical visit. Clinical events of interest throughout the entire follow-up period were derived from in-patient clinical records at all 3 centers, the National Patient Overview at the 2 Swedish centers, as well as annual reports from the Transplant Registry in Gothenburg. Immunological data on recipients and basic data on kidney donors were retrieved from the Scandiatransplant Registry.

From the original SAILOR study database, the following data were extracted: demographic data at baseline, treatment arm assignment, tacrolimus trough levels within the first 2 years, 1- and 2-year measured glomerular filtration rate (mGFR), diagnosis of PTDM, incidence of donor-specific antibodies (DSAs) at 1 year, central and blinded assessment of kidney transplant histopathological diagnoses from protocol biopsies at 1 year, and for-cause transplant biopsies within 2 years after transplantation.

For-cause transplant biopsies beyond 1 year were captured from clinical records and the histopathological database. Human leukocyte antigen–antibody testing after 1 year was performed according to each center’s practice and captured from clinical records as well as the human leukocyte antigen laboratory database.

### Diagnostics

Diagnosis of PTDM beyond 2 years was determined by each center’s practice based on the American Diabetes Association criteria, fasting plasma glucose or glycated hemoglobin, however, without performing oral glucose tolerance testing.

Kidney function was assessed using eGFR at 1, 2, and 5 years, and the last follow-up, based on creatinine measurements, using the following 4 different equations: (i) Modification of Diet in Renal Disease, (ii) Lund-Malmö equation, (iii) Chronic Kidney Disease Epidemiology Collaboration, and (iv) race-free Chronic Kidney Disease Epidemiology Collaboration 2021.^11^, ^12^, ^13^, ^14^ GFR estimates were correlated with GFR measurements (obtained by iohexol clearance), which were performed according to protocol in the original RCT and subsequently according to each center’s practice.

Transplant biopsies were assessed according to the Banff 2017 diagnostic criteria. The for-cause biopsies during the 2 years after transplantation, and 1-year protocol biopsies were assessed centrally and blindly at the center in Gothenburg by 2 pathologists independently. Transplant biopsies beyond 2 years were assessed by pathologists at each center.

### End Points

The efficacy end points were as follows: (i) patient survival, (ii) death-censored graft survival, (iii) overall graft survival, (iv) incidence of biopsy-proven rejection, (v) incidence of PTDM, and (vi) eGFR.

The safety end points were incidence of the following: (i) severe infections (including opportunistic infections) requiring hospitalization, (ii) opportunistic infections not requiring hospitalization, (iii) malignancies (including nonmelanoma skin cancer), (iv) major cardiovascular events (myocardial infarction, percutaneous coronary intervention, coronary artery bypass graft surgery, stroke, heart failure requiring hospitalization, or cardiovascular death), and (v) DSA.

### Statistical Analysis

In this retrospective study, all analyses were descriptive for the intention-to-treat population, whereas only the cumulative incidence of PTDM was calculated for the per-protocol population of the original SAILOR RTC. Comparisons were made between the SA arm and the SM arm. Statistical significance was set at a 2-sided *P*-value < 0.05.

Continuous data were summarized as means with SDs, categorical data were presented in counts or percentages, and time-to-event end points were calculated from the date of kidney transplantation to the event. For comparison between the 2 groups, Fisher exact test for dichotomous variables, Mantel-Haenszel chi-square test for categorical variables, and *t* test for continuous variables were used. Time-to-event end points were analyzed with the Kaplan-Meier method. The calculation of confidence intervals (CI) for continuous variables is based on the assumption of normality. Cox regression analysis was used to identify risk factors and calculate adjusted hazard ratios (aHRs) for events. Adjusted models were made by the stepwise selection of independent covariates; the level of significance was 0.05.

Differences between mGFR and the 4 eGFR equations were tested with the Wilcoxon signed-rank test. Intraclass correlation coefficient was calculated with the Shrout-Fleiss reliability random set. The statistical software SAS, version 9.4 TS level 1M6 (Cary, NC) was used for the analyses.

## Results

**Figure 1:**
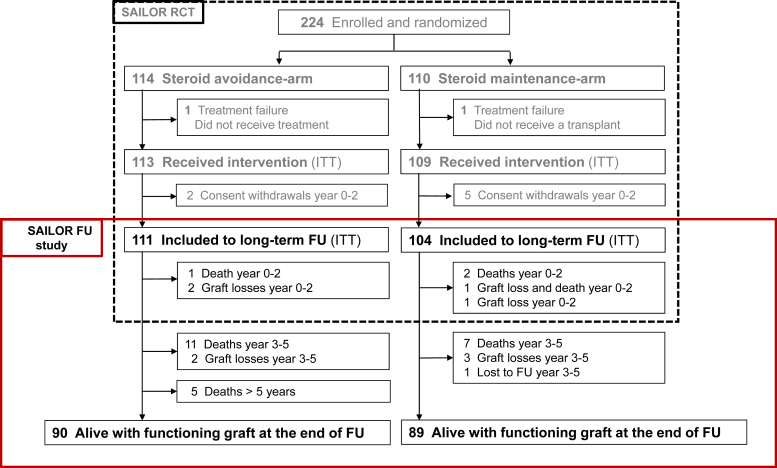
Study flow chart and patient disposition. FU, follow-up; ITT, intention-to-treat population.

Of the 222 participants in the original SAILOR study, 7 withdrew their consent, and the remaining 215 were included in this follow-up study. Of these, 111 patients were originally randomized to the SA arm, and 104 patients to the SM arm (Figure 1).

Baseline characteristics were comparable between the 2 study arms and were in line with the original study cohort (Table 1). As reported previously, the tacrolimus trough levels were comparable in both treatment arms at all time points up to 2 years, with all mean levels < 8 μg/l, except at 1-week posttransplant week when the level was significantly higher in the SA arm than in the SM arm (11.8 vs. 9.9 μg/l, *P* = 0.003).^9^ The mean follow-up time was 7.3 ± 1.8 years for participants in the SA arm and 7.3 ± 2.0 years in the SM arm (Table 2). At the end of the follow-up, 179 study participants (80.6%), 90 in the SA arm and 89 in the SM arm, were alive with functioning grafts (Figure 1).

**Table 1:** Demographics

Variable	SAILOR RCT	SAILOR FOLLOW-UP		
	(Total *N* = 222)	Steroid avoidance + ATG (*n* = 111)	Steroid maintenance + basiliximab (*n* = 104)	*P*-value^a^
Age at Tx	50.7 ± 14.2	52.6 ± 13.7	48.9 ± 14.7	0.061
Age > 60 yrs	61 (27.5)	34 (30.6)	26 (25.0)	0.44
Female sex	61 (27.5)	32 (28.8)	28 (26.9)	0.87
BMI	26.1 ± 3.9	25.9 ± 4.0 *n* = 108	26.1 ± 3.9 n=103	0.69
Living donor	91 (41.0)	51 (45.9)	46 (44.2)	
Living donor age	-	49.9 ± 10.8 *n* = 43	49.3 ± 11.1 *n* = 39	
Deceased donor	131 (59.0)	60 (54.1)	58 (55.8)	0.91
Deceased donor age	-	57.6 ± 15.9 *n* = 63	55.6 ± 16.2 *n* = 60	
Number of HLA mismatches (A/B/DR)	1,1/1,3/1,2	1.1/1.3/1.2	1.1/1.4/1.2	
Cause of ESRD				
Glomerulonephritis	66 (29.7)	34 (30.6)	31 (29.8)	
Sec. glomerulonephritis	3 (1.4)	1 (0.9)	2 (1.9)	
Interstitial nephritis or pyelonephritis	10 (4.5)	7 (6.3)	2 (1.9)	
Polycystic kidney disease	70 (31.5)	36 (32.4)	31 (29.8)	
Hypertension or large vessel disease	18 (8.1)	10 (9.0)	8 (7.7)	
Hereditary or congenital kidney disease	13 (5.9)	4 (3.6)	9 (8.7)	
Others	10 (4.5)	4 (3.6)	2 (1.9)	
Undefined cause	32 (14.4)	15 (13.5)	19 (18.3)	

ATG, antithymocyte globulin; BMI, body mass index; ESRD, end-stage renal disease; HLA, human leukocyte antigen; RCT, randomized controlled trial; SA, steroid avoidance; SM, steroid maintenance; Tx, transplantation.

Mean ± SD, *n* (%)

a Comparison between SA and SM in the follow-up study.

**Table 2:** Efficacy end points

Variables	Steroid avoidance + ATG *n* = 111	Steroid maintenance + basiliximab *n* = 104	*P*-value
FU time (yrs)	7.3 ± 1.76	7.3 ± 2.03	0.99
Patient survival, 7 yrs	0.877 (0.791–0.929)	0.931 (0.860–0.966)	0.32
Age at death	62.0 ± 9.6	62.6 ± 9.6	0.88
Time from Tx to death (mos)	74.2 ± 23.1	54.7 ± 38	<0.0001
Deaths, all causes	17 (15.3)	10 (9.6)	0.22
Cardiovascular	6 (5.4)	0	0.03
Malignancy	8 (7.2)	2 (1.9)	0.1
Infection-related	1 (0.9)	5 (4.8)	0.1
Other	2 (1.8)	3 (2.9)	0.67
Death-censored graft survival 7 yrs	0.918 (0.841–0.959)	0.931 (0.860–0.966)	0.88
Graft loss, all causes	7 (6.3)	5 (4.8)	0.77
Rejection	3 (2.7)	3 (2.9)	1.0
Recurrence of glomerular disease	1 (0.9)	1 (1.0)	1.0
Primary nonfunction	1 (0.9)	0	1.0
Other	2 (1.8)	1 (1.0)	1.0
Patient and graft survival7 yrs	0.813 (0.721–0.877)	0.893 (0.815–0.939)	0.19
Recurrence of glomerulonephritis	5 (4.5)	6 (5.8)	0.76
BKVN	6 (5.4)	1 (1.0)	0.12
*De novo* DSA (anytime)	15 (13.5)	16 (15.4)	0.70

ATG, antithymocyte globulin; BKVN, BK virus nephropathy; CI, confidence interval; DSA, donor-specific antibody; FU, follow-up; Tx, transplantation.

Mean ± SD; survival (CI); *n* (%).

### Efficacy End Points

The data for efficacy end points are presented in Table 2.

#### Patient Survival

**Figure 2:**
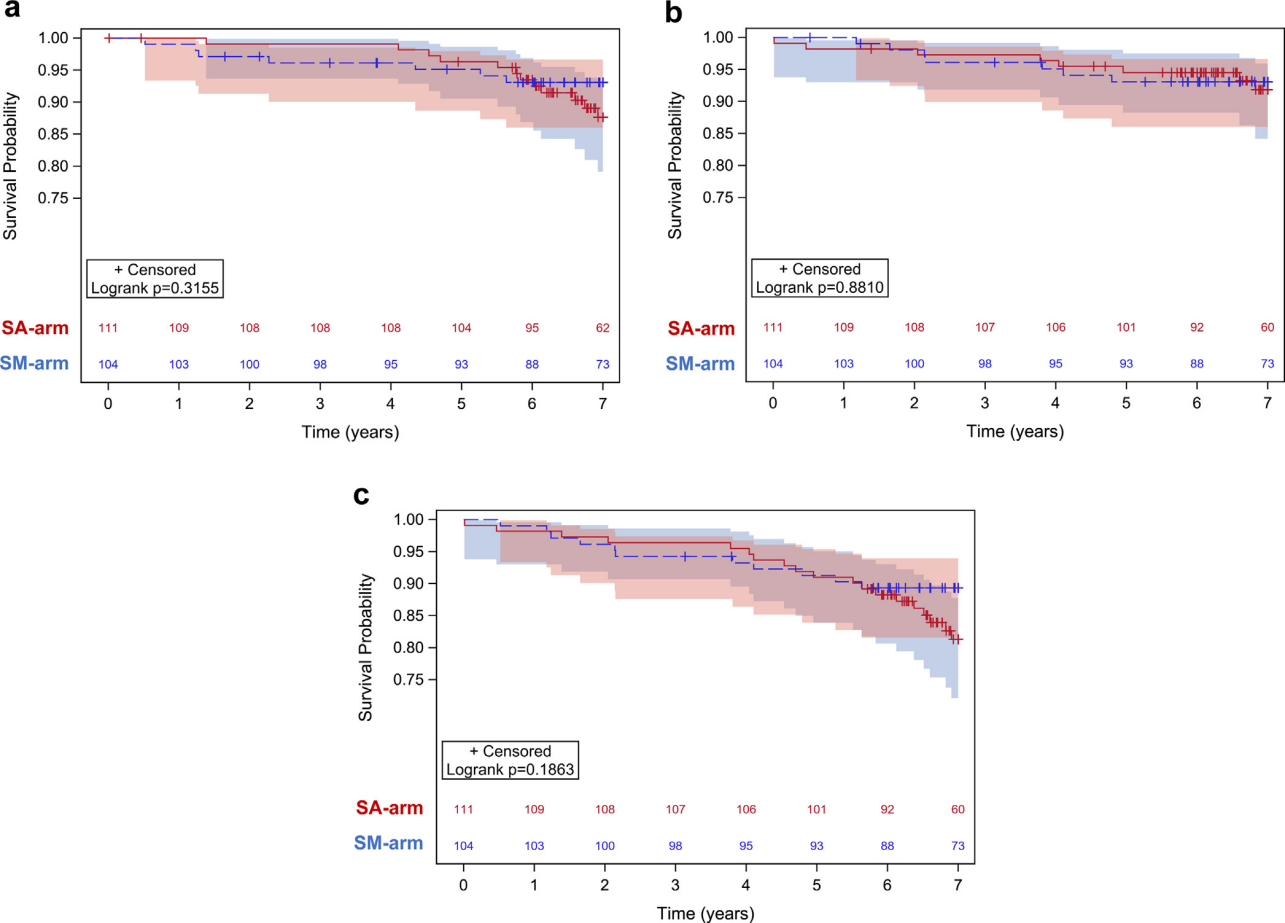
(a). Patient survival according to study arm. (b) Death-censored graft survival according to study arm. (c) Overall graft survival according to study arm. SA, steroid avoidance; SM, steroid maintenance.

Patient survival was comparable between the 2 arms as follows: 88% (95% CI: 0.79–0.93) in the SA arm versus 93% (95% CI: 0.86–0.97) in the SM arm (Table 2, Figure 2a and Supplementary Table S1). Seventeen deaths were observed in the SA arm (15.3%) and 10 (10.6%) in the SM arm (*P* = 0.22); the cause of death was most commonly cardiovascular disease and malignancy. The mean time from transplantation to death was longer in the SA arm (74.2 ± 23.1 months) than in the SM arm (54.7 ± 38.1 months) (*P* < 0.0001). Multivariate Cox regression analyses revealed DSA at 1 year as a risk factor with an adverse impact on patient survival (aHR: 11 [95% CI: 1.31–91.7], *P* = 0.027), in addition to recipient age at transplant (aHR: 1.10 [95% CI :1.05–1.14], *P* < 0.0001) (Table 3).

**Table 3:** Cox analysis for patient survival and death-censored graft survival

Risk factors	Univariable		Multivariable	
Patient survival	HR (95% CI)	*P*-value	HR (95% CI)	*P*-value
Treatment group	0.62 (0.28–1.36)	0.23		
Age at Tx	1.09 (1.05–1.14)	<.0001	1.10 (1.05–1.14)	<.0001
Gender	1.17 (0.51–2.67)	0.72		
Donor age	1.05 (1.02–1.09)	0.002		
PTDM at 1 yr	1.98 (0.75–5.23)	0.17		
Rejection at 1 yr	1.25 (0.38–4.16)	0.72		
DSA	5.07 (1.17–21.9)	0.03	11.0 (1.31–91.7)	0.03
Death-censored graft survival				
Treatment group	0.93 (0.34–2.55)	0.88		
Age at Tx	1.00 (0.96–1.04)	0.99		
Gender	1.79 (0.64–5.03)	0.27		
Donor age	1.04 (1.00–1.08)	0.08		
PTDM at 1 yr	1.22 (0.28–5.40)	0.79		
Rejection at 1 yr	3.76 (1.20–11.8)	0.02	3.76 (1.20–11.8)	0.02
DSA	3.14 (0.41–23.9)	0.27		

CI, confidence interval; DSA, donor-specific antibody; HR, hazard ratio; PTDM, posttransplantation diabetes mellitus; Tx, transplantation.

#### Graft Survival

In the intention-to-treat analysis, death-censored graft survival was similar in the 2 arms: 92% (95% CI: 0.84–0.96) in the SA arm versus 93% (95% CI: 0.86–0.97) in the SM arm (Table 2, Figure 2b and Supplementary Table S2). Graft loss was observed in a total of 12 participants: 7 in the SA arm and 5 in the SM arm. Three grafts in each arm were lost due to rejection. The overall graft survival was 81% (95%: CI: 0.72–0.88) in the SA arm versus 89% (95% CI: 0.82–0.94) in the SM arm, which was also not significantly different (Figure 2c and Supplementary Table S3). Graft loss from any cause (including death), analyzed with multivariate Cox regression, was adversely affected by any experienced rejection up to 1-year posttransplant (aHR 3.76 [95% CI: 1.2–11.81], *P* = 0.023) (Table 3).

#### Biopsy-Proven Rejection and DSA

**Figure 3:**
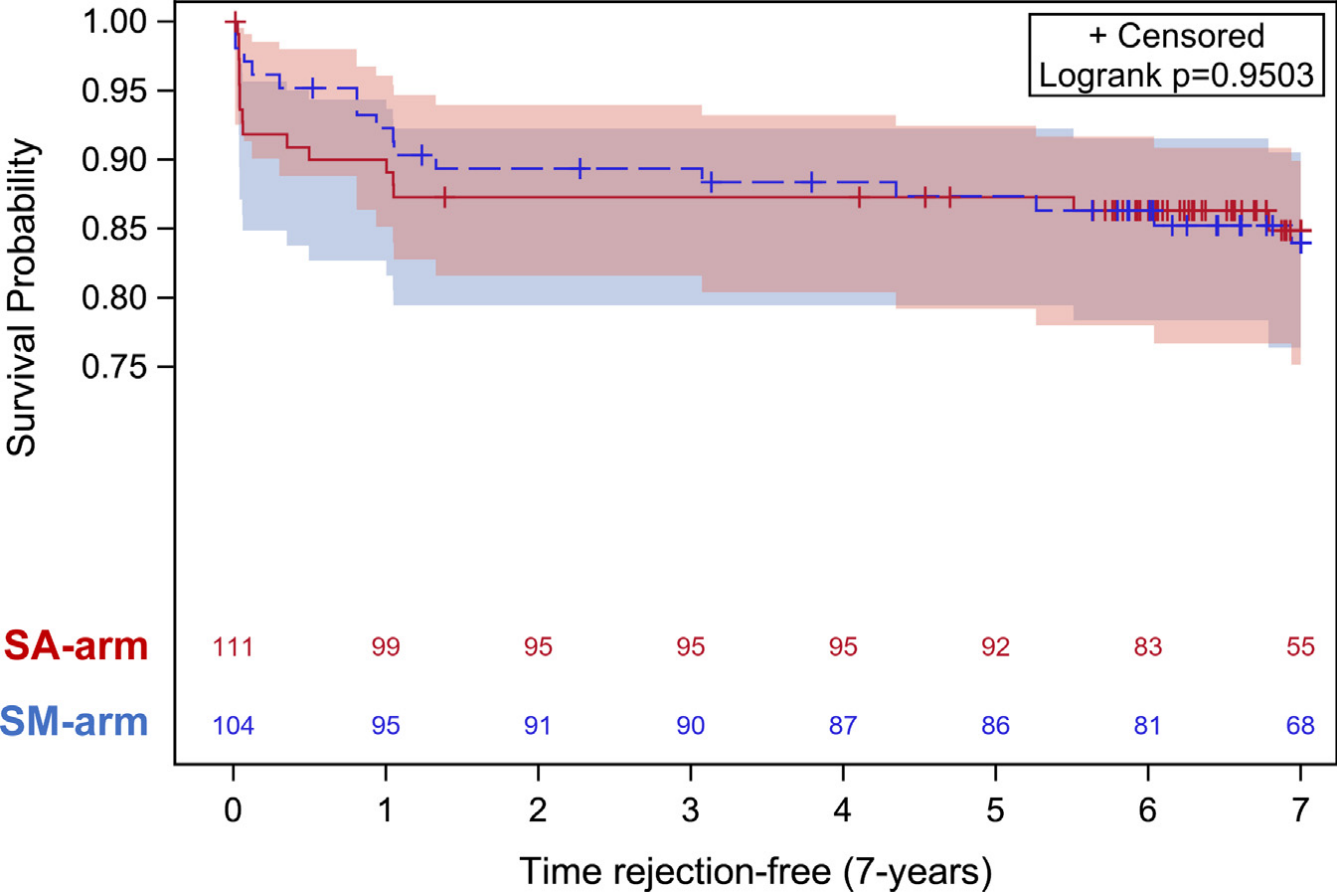
Rejection-free survival according to study arm. SA, steroid avoidance; SM, steroid maintenance.

The cumulative incidence of biopsy-proven rejection from transplantation to the last follow-up was similar in the 2 arms: 18% in the SA arm versus 15.4% in the SM arm (*P* = 0.72) (Table 4). Biopsy-proven rejection–free survival was also similar: 85% (95% CI: 0.764–0.905) in the SA arm versus 84% (95% CI: 0.752–0.899) in the SM arm (Figure 3 and Supplementary Table S4). The types of rejection, that is, active or acute, or chronic, and T-cell–mediated or antibody-mediated, were distributed similarly in the 2 treatment arms (Table 4). The incidence of *de novo* DSA also did not differ between the SA and SM arms (13.5 vs. 15.4%, *P* = 0.70) (Table 2).

**Table 4:** Biopsy-proven rejections

Rejection type	Steroid avoidance + ATG *n* = 111	Steroid maintenance + basiliximab *n* = 104	*P*-value
Patient with any BPR	20 (18.0)	16 (15.4)	0.72
BPR episodes (n)	28	22	0.52
aABMR	1	3	0.31
aABMR + aTCMR	-	2	0.19
aTCMR (Banff 1 / Banff 2 ± Banff1)	13 (8 / 5)	5 (2 / 3)	0.14
aTCMR + cABMR	1	-	1.0
aTCMR + cTCMR	1	1	1.0
ca ABMR	5	1	0.21
ca ABMR + cTCMR	-	1	0.44
cABMR	2	1	1.0
cTCMR (Banff 1 / Banff 2 ± Banff1)	5 (3 / 2)	8 (4 / 4)	0.2
Active / acute BPR	16	11	0.78
Chronic BPR	12	11	0.78
TCMR	19	14	0.77
ABMR	8	5	0.75
Mixed ABMR + TCMR	1	3	0.31
Subclinical rejection (in protocol biopsy)	7	9	0.36
Clinical rejection (in for-cause biopsy)	21	13	0.36
Rejection within year 1 / all rejections	18 / 28	17 / 22	0.37

aABMR, active antibody-mediated rejection; aTCMR, active/acute T-cell–mediated rejection; ATG, antithymocyte globulin; BPR, biopsy-proven rejection; caABMR, chronic active antibody-mediated rejection; cABMR, chronic antibody-mediated rejection; cTCMR, chronic T-cell–mediated rejection.

*n* (%).

#### Kidney Function

**Figure 4:**
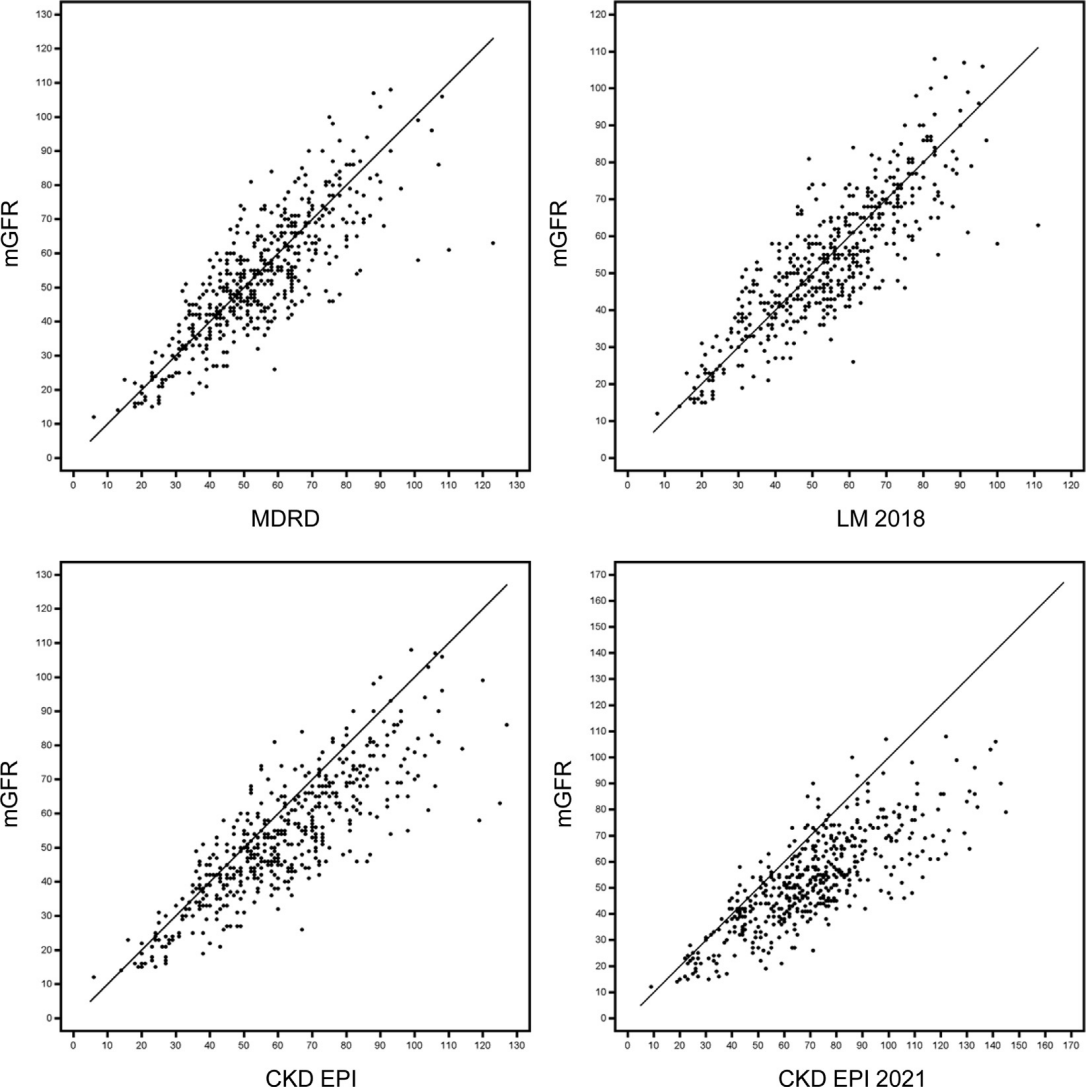
Correlation between eGFR and mGFR (4 equations). CKD-EPI, Chronic Kidney Disease Epidemiology Collaboration; CKD-EPI 2021, CKD-EPI race-free eGFR equation; eGFR, estimated glomerular filtration rate (ml/min per 1.73 m^2^); LM, Lund-Malmö; MDRD, Modification of Diet in Renal Disease; mGFR, measured glomerular filtration rate (ml/min per 1.73 m^2^).

There were no significant differences in eGFR between the SA and SM arms at any time point (Table 5). A total of 508 available mGFR measurements (iohexol clearance), 394 from the original SAILOR RCT and 114 from the current study were correlated intraindividually with eGFR values estimated using 4 different equations (Modification of Diet in Renal Disease, Chronic Kidney Disease Epidemiology Collaboration, Chronic Kidney Disease Epidemiology Collaboration 2021, and Lund-Malmö equation) (Table 6).^11^, ^12^, ^13^, ^14^ The Modification of Diet in Renal Disease equation showed good correlation with mGFR in our cohort (intraclass correlation coefficient: 0.82) and was thus used to report eGFR in this study (Figure 4).

**Table 5:** Kidney function as assessed by eGFR using MDRD equation

Visit	Steroid avoidance + ATG	Steroid maintenance + basiliximab	Difference between groups (95% CI)	*P*-value
Year 1	*n* = 110	*n* = 104		
	53.6 (18.8) *n* = 109	53.6 (16.1) *n* = 103	0.070 (−4.678 to 4.818)	0.98
Year 2	*n* = 109	*n* = 103		
	52.6 (19.1) *n* = 109	54.5 (17.5) *n* = 100	−1.98 (−6.99 to 3.04)	0.44
Year 5	*n* = 106	*n* = 97		
	54.7 (19.8) *n* = 101	55.7 (17.4) *n* = 93	−0.943 (−6.248 to 4.362)	0.73
End of FU	*n* = 94	*n* = 90		
	53.8 (21.6) *n* = 93	56.1 (18.2) *n* = 88	−2.29 (−8.16 to 3.59)	0.44
Last recorded	*n* = 111	*n* = 104		
visit	50.8 (22.3) *n* = 109	54.0 (19.7) *n* = 103	−3.22 (−8.93 to 2.48)	0.27

ATG, antithymocyte globulin; BMI, body mass index; eGFR, estimated glomerular filtration rate (ml/min per 1.73 m^2^); FU, follow up; MDRD, Modification of Diet in Renal Disease eGFR equation^11^; mGFR, measured glomerular filtration rate (ml/min per 1.73 m^2^).

Mean (±SD) / n = is presented. For comparison between groups, a *t* test was used.

**Table 6:** Correlation between eGFR and mGFR

eGFR equation formula	Difference eGFR − mGFR	*P*-value	CV %	II SD	ICC
MDRD	1.38 (−19.45 to 22.20) (10.62) *n* = 507	0.0178	14.11	7.57	0.815
CKD-EPI	8.42 (−13.63 to 30.48) (11.25) *n* = 507	< 0.0001	17.17	9.93	0.754
CKD-EPI 2021	17.8 (−11.6 to 47.2) (15.0) *n* = 486	< 0.0001	25.98	16.45	0.558
LM 2018	1.09 (−18.46 to 20.65) (9.98) *n* = 507	0.0291	13.29	7.09	0.840

CKD-EPI, Chronic Kidney Disease Epidemiology Collaboration; CKD-EPI 2021, race-free eGFR equation; CV, coefficient of variance (Intraindividual SD ∗ 100 / mean); eGFR, estimated glomerular filtration rate (ml/min per 1.73 m^2^); ICC, intraclass correlation coefficient (Shrout-Fleiss reliability: random set); II SD, intraindividual SD; LM 2018, Lund-Malmö; MDRD, Modification of Diet in Renal Disease; mGFR, measured glomerular filtration rate (ml/min per 1.73 m^2^).

For the difference between eGFR-mGFR, mean (95% CI, Limits of Agreement) / (SD) / and *n* are presented.

### Safety End Points

The data for safety end points are presented in Table 7. The cumulative incidence of PTDM in the intention-to-treat population at the end of follow-up was 22.5% in the SA arm versus 27.9% in the SM arm (*P* = 0.43). Eighteen new cases of PTDM were observed after a 2-year visit: 10 in the SA arm and 8 in the SM arm. In the per-protocol population, the cumulative incidence of PTDM was 14.4% in the SA arm, which was significantly lower than the 27.9% in the SM arm (*P* = 0.008).

**Table 7:** Safety end points

Variables (subjects with event)	Steroid avoidance + ATG *n* = 111	Steroid maintenance + basiliximab *n* = 104	*P*-value
PTDM ITT	25 (22.5)	29 (27.9)	0.43
PTDM PP	16 (14.4)	29 (27,9)	0.019
Serious infections requiring hospitalization	63 (56.8)	61 (58.7)	0.78
Serious infections requiring hospitalization / patient	1.46 ± 2.11	1.46 ± 2.34	
> 1 serious infection requiring hospitalization	33 (29.7)	37 (35.6)	0.38
Bacterial	34 (30.6)	30 (28.8)	0.88
Bacterial and viral	1 (0.9)	1 (1.0)	1.00
CMV	7 (6.3)	5 (4.8)	0.77
Other viral	14 (12.6)	13 (12.5)	1.00
Agent not specified	40 (36.0)	44 (42.3)	0.40
GI infection	18 (16.2)	19 (18.3)	0.72
Meningitis/encephalitis		2 (1.9)	0.23
Other infections, including fever and unknown origin	22 (19.8)	21 (20.2)	1.00
Sepsis	6 (5.4)	9 (8.7)	0.43
Urinary tract infection	30 (27.0)	21 (20.2)	0.26
Pneumonia	15 (13.5)	18 (17.3)	0.46
Skin infection	10 (9.0)	5 (4.8)	0.29
Opportunistic infection NOT requiring hospitalization	19 (17.1)	24 (23.1)	0.31
BK viremia	10 (9.0)	12 (11.5)	0.65
CMV	2 (1.8)	10 (9.6)	0.02
Varicella zoster infection	6 (5.4)	3 (2.9)	0.65
Other	1 (0.9)	1 (1.0)	1,00
Malignances	37 (33.3)	32 (30.8)	0.77
PTLD	3 (2.7)	0	0.25
Central nervous system	0	1 (1.0)	0.48
Gastro-intestinal	3 (2.7)	3 (2.9)	1.00
Hematological	2 (1.8)	0	0.50
Non-melanoma skin ca	25 (22.5)	25 (24.0)	0.87
Respiratory	2 (1.8)	2 (1.9)	1.00
Urogenital	4 (3.6)	2 (1.9)	0.68
Other	3 (2.7)	1 (1.0)	0.62
MACE	16 (14.4)	9 (8.7)	0.21
Immunosuppression at last follow-up	*n* = 95	*n* = 90	
Steroids	33 (34.7)	77 (85.6)	<0.001
Tacrolimus	90 (94.7)	83 (92.2)	0.69
MMF	84 (88.4)	84 (93.3)	0.37
mTOR	3 (2.7)	1 (1.0)	0.67
Cyclosporine	2 (2.1)	3 (3.3)	0.95
Other	7 (7.4)	7 (7.8)	1.00

ATG, antithymocyte globulin; ca, cancer; CMV, cytomegalovirus; FU, follow-up; GI, gastrointestinal; ITT, intention-to-treat population; MACE, major cardiovascular events; MMF, mycophenolate mofetil; mTOR, mammalian target of rapamycin; PP, per-protocol population; PTDM, posttransplantation diabetes mellitus; PTLD, posttransplantation lymphoproliferative disorder.

Mean ± SD; no. (%).

Patients with serious infections requiring hospitalization over the entire observation period were equally distributed in the 2 arms: 56.8% in the SA arm and 58.7% in the SM arm (*P* = 0.78). Cytomegalovirus disease requiring hospitalization was similar in the 2 arms, but we observed a lower rate of cytomegalovirus infections (cytomegalovirus viremia, cytomegalovirus syndrome) not requiring hospitalization in the SA arm (1.8% vs. 9.6%, *P* = 0.02). The incidence of all other opportunistic infections that did not require hospitalization were similar in the 2 arms.

The incidence of malignancy did not differ between treatment arms: 17.1% in the SA arm versus 23.1% in the SM arm (*P* = 0.77). Malignancy, as a cause of death, tended to dominate in the SA arm, 9.0% versus 1.9% in the SM arm; however, the observed difference did not reach statistical significance.

The incidence of major cardiovascular events was 14.4% in the SA arm versus 8.7% in the SM arm (*P* = 0.21). Cardiovascular cause of death was observed in 6 recipients (5.4%) in the SA arm and none in the SM arm (*P* = 0.03).

In the SA arm, 33 participants (34.7%) were started on steroids, 62 (65.3 %) remained on steroid-free immunosuppression; whereas in the SM arm, 13 participants(14.4 %) had steroids withdrawn at the end of the follow-up (Table 7).

## Discussion

The SAILOR RCT compared SA with ATG induction and SM with basiliximab induction among kidney transplant recipients with low-immunogenic risk. Low-dose tacrolimus and MMF were used in both arms. The RCT did not demonstrate a lower incidence of PTDM within the first 2 posttransplant years in the SA arm, in contrast to a few other studies.^8^^,^^15^ At the same time, the incidence of overall biopsy-proven rejection (acute or chronic) was similar in the 2 arms.^9^

The current observational study, SAILOR FU, reports long-term outcomes for participants included in the original RCT. The extended follow-up data indicate that the SA regimen following ATG induction is efficient and safe 7 years after transplantation. Although the study was not designed or powered to prove differences in robust efficacy end points, it showed similar proportions of graft survival, patient survival, incidence of biopsy-proven rejection, and kidney function in the SA arm as compared with the SM arm. Furthermore, safety parameters, such as the incidence of PTDM, serious infections requiring hospitalization, major cardiovascular events, and malignancy were similar in the 2 treatment arms. However, the cumulative incidence of PTDM was significantly lower in SA arm when calculated for the per-protocol population. Surprisingly, more cardiovascular deaths were observed in the SA arm, which may be linked to the higher mean age at inclusion in the SA arm (by 3.7 years) compared with the SM arm; a difference that could have been a chance finding.

Even with an extended follow-up period of approximately 7 years, our findings are consistent with those of the Harmony FU study, which demonstrated excellent efficacy and safety at 5 years posttransplant. The 5-year death-censored graft survival rate was 94.9% in the SM (control) arm, compared with 93% and 91.4% in the 2 rapid SWD arms featuring ATG and basiliximab, respectively.^10^ However, contrary to the Harmony FU study, which identified rapid SWD as a significant independent positive factor for patient survival (aHR: 0.55), we did not observe such an effect of treatment modality in our patients. It is important to note that none of the participants in the SAILOR study had diabetes before transplantation, whereas 15% of Harmony study participants did, which may have contributed to the greater benefit from early SWD observed in the Harmony FU study.

To date, the only long-term RCT, designed with a 5-year follow-up, was the Corticosteroid Cessation Study.^16^ This double-blind, placebo-controlled, multicenter trial included 386 kidney transplant recipients who received treatment with standard-dose tacrolimus + MMF + antibody induction and either early SWD or continued steroids. The study found no significant difference in the proportion of patients reaching the primary composite end point of death, graft loss, or moderate or severe acute rejection, with rates of 15.7% for early SWD compared with 14.4% for continued steroids (*P* = 0.69). Although early SWD was associated with a higher biopsy-proven acute rejection compared with continued steroids (*P* = 0.04), the regimen was associated with improved cardiovascular risk factors, including triglycerides, PTDM requiring insulin, and weight. Notably, this study’s population differs substantially from those in SAILOR FU as well as Harmony FU in several key aspects, including younger recipient age, race (approximately 20% African Americans), possibly higher immunological risk with accepted peak panel reactive antibodies < 49%, higher tacrolimus trough levels (10–20 ng/ml in first 3 months, thereafter 5–15 ng/ml), and the exclusion of recipients with delayed graft function. Recently, a 15.8-year follow-up of this study reported similar overall graft survival and death-censored graft survival in the early SWD arm and the continued steroids arm (aHR: 0.83 and 0.78, respectively). The authors concluded that long-term corticosteroids may not be a necessary component of a calcineurin inhibitor–based multidrug immunosuppressive regimen.^17^

Other long-term studies on this topic are observational, which together with the studies highlighted herein, are summarized in Table 8.^2^^,^^6^^,^^8^^,^^10^^,^^16^, ^17^, ^18^, ^19^ The ADVANCE FU study recently reported 5-year results for the original 24-week RCT, which compared early SWD with SA + basiliximab induction, prolonged-release standard-dose tacrolimus, and MMF.^19^ Graft and patient survival rates were numerically high and did not differ significantly between the 2 treatment arms, despite a higher incidence of biopsy-proven acute rejection in the SA arm at 6 months. Notably, the ADVANCE study did not include an SM comparator arm. Likewise, the ATLAS FU trial showed no significant difference in 3-year patient or graft survival among patients receiving basiliximab + standard tacrolimus monotherapy, standard tacrolimus + MMF, and standard triple therapy without antibody induction (93.1%, 96.4%, and 97.0% for patient survival; 92.7%, 92.5%, and 92.5% for graft survival, respectively), despite a higher incidence of early biopsy-proven acute rejection in the 2 steroid-free arms at 6 months (26.1%, 30.5%, and 8.2%, respectively; *P* < 0.001).^18^

**Table 8:** Overview of steroid-weaning RCTs with long-term FU (utilizing standard or low-dose tacrolimus)

Name of study	N time orig. study time FU study	Immunosuppression	Main study findings	FU study findings
Atlas Vítko *et al.*, 2005^2^ Krämer *et al.*, 2012^18^	*n* = 451 / 421 6 mos 3 yrs	1. IL-2Ri + standard-tac 2. Standard-tac, MMF 3. Standard-tac, MMF, CS	Lowest BPAR in arm 3	Similar BPAR 6m-3 yr, graft survival and patient survival
ADVANCE Mourad *et al.*, 2017^6^ Pernin *et al.*, 2023^19^	*n* = 1081 / 772 24 wks 5 yrs	1. IL-2Ri + standard-tac, MMF, ESWD 2. IL-2Ri + standard-tac, MMF	Similar PTDM; Higher BPAR in arm 2	Similar graft survival and risk of graft loss and death
Harmony Thomusch *et al.*, 2016^8^ Stumpf, 2024^10^	*n* = 615 / 359 1 yr 5 yrs	A. IL-2Ri + low-tac, MMF, CS B. IL-2Ri + low-tac, MMF, ESWD C. ATG + low-tac, MMF, ESWD.	Similar, low BPAR; Lower PTDM in arm B and C vs. arm A	Low BPAR and death censored-graft loss independent of ESWD; ESWD positive factor for patient survival
CS Cessation Woodle et al., 2008^16^ Woodle et al., 2021^17^	n= 385 5 years 15.8 years	1. ATG / IL-2Ri + standard-tac, MMF, ESWD 2. ATG / IL-2Ri + standard-tac, MMF, CS	Similar proportion of death / graft loss / BPAR; Higher BPAR in arm 1	Similar aHR for graft failure from any cause

ATG, antithymocyte globulin; BPAR, biopsy-proven acute rejection; CS, corticosteroids; ESWD, early steroid withdrawal; FU, follow-up; IL-2Ri, interleukin-2 receptor inhibitor; MMF, mycophenolate mofetil; PTDM, post-transplantation diabetes mellitus; pr-tac, prolonged release tacrolimus; RCT, randomized controlled trial; tac, tacrolimus.

In our study, SAILOR FU, biopsy-proven rejection rates (any rejection) were 19.8% in the SA arm compared with 16.3% in the SM arm (*P* = 0.6). These rates are slightly higher than those reported in other studies, such as 17.8% versus 10.8% for early SWD versus CCS in the Early Corticosteroid Cessation study, and 14.7% versus 14.5% versus 13.2% for SM-basiliximab versus rapid SWD-basiliximab versus rapid SWD-ATG in the Harmony FU study. This difference may be attributed to protocol biopsies at 1 year being performed in most patients in the SAILOR study, which probably contributed to higher detection of rejections. Moreover, the mean follow-up time was longer in the SAILOR FU study, 7.3 years, than in the Early Corticosteroid Cessation study and the Harmony FU study, both 5 years. In addition, our study included participants with delayed graft function, who are known to be at higher risk of acute rejection and graft failure when early SWD is implemented.^20^

We did not find significant differences in the prevalence of *de novo* DSA or chronic rejections between the SA and the SM arms. Notably, multivariate regression analysis revealed that the presence of DSA within 1 year of transplantation negatively impacted long-term patient survival, and any rejections within 1 year negatively affected long-term graft survival. In contrast, treatment arm assignment was not identified as a risk factor for either outcome.

The strengths of our study include a long mean follow-up period of 7.3 years, a high retention rate, with approximately 70% of participants maintaining their original assignment to either the SA or the SM arm, and the assessment of kidney function using the Modification of Diet in Renal Disease eGFR formula that correlated well with mGFR. The main limitation of the study is its observational design, which lacks a statistical power calculation for robust end points, such as graft survival and patient survival. Moreover, oral glucose tolerance testing was not used for the diagnosis of PTDM in the follow-up period. Conclusions are only applicable to a specific patient population of Caucasian origin with low immunologic risk. In fact, the association of early SWD with transplant outcomes has been less favorable in recipients with higher panel reactive antibodies, particularly those with panel reactive antibodies > 60%.^21^

In conclusion, the SAILOR FU study provides further support for the tailored use of SA for selected kidney transplant recipients, demonstrating sustained safety even in the long-term. Consistent with findings of other recent studies, we suggest that steroid-weaning strategies, with the current regimen using low-dose tacrolimus + MMF, following antibody induction, could benefit more patients than currently recognized. Still, it is crucial to carefully select eligible kidney transplant recipients to ensure they can fully benefit from avoiding the adverse effects of long-term steroid use.

## Disclosure

All the authors declared no competing interests.

## Supplementary Material

Supplementary File (PDF) **Table S1.** Risk table for patient survival up to 7 years according to treatment arm. **Table S2.** Risk table for graft survival (death-censored) up to 7 years according to treatment arm. **Table S3.** Risk table for overall graft survival up to 7 years according to treatment arm. **Table S4.** Risk table for rejection-free survival up to 7 years according to treatment arm.

## Footnotes

Supplementary File (PDF)
**Table S1.** Risk table for patient survival up to 7 years according to treatment arm. **Table S2.** Risk table for graft survival (death-censored) up to 7 years according to treatment arm. **Table S3.** Risk table for overall graft survival up to 7 years according to treatment arm. **Table S4.** Risk table for rejection-free survival up to 7 years according to treatment arm.
