# Supplementary material for: Steroid Avoidance With Low-Dose Tacrolimus is Safe and Effective in the Long-Term for Kidney Transplant Recipients
Source: Kidney Int Rep. 2025 Jun 19;10(9):3102–12. doi: 10.1016/j.ekir.2025.06.016 (PMC12446927; doi:10.1016/j.ekir.2025.06.016)
Supplement: Supplementary File (PDF) — Table S1. Risk table for patient survival up to 7 years according to treatment arm. Table S2. Risk table for graft survival (death-censored) up to 7 years according to treatment arm. Table S3. Risk table for overall graft survival up to 7 years according to treatment arm. Table S4. Risk table for rejection-free survival up to 7 years according to treatment arm. [file mmc1.pdf]

Supplementary Table S1. Risk table for patient survival up to 7 years according to study arm

**Time** (years)	**Survival Probability**	
	**Steroid avoidance + ATG**	**Steroid maintenance + IL2**
1	1.000	0.990
2	0.991	0.971
3	0.991	0.961
4	0.991	0.961
5	0.963	0.951
6	0.935	0.931
7	0.877	0.931

Supplementary Table S2. Risk table for graft survival (death-censored) up to 7 years according to study arm

Time (years)	Survival Probability	
	**Steroid avoidance + ATG**	**Steroid maintenance + IL2**
1	0.982	1.000
2	0.982	0.980
3	0.973	0.961
4	0.964	0.951
5	0.945	0.931
6	0.945	0.931
7	0.918	0.931

Supplementary Table S3. Risk table for overall survival up to 7 years according to study arm

Time (years)	Survival Probability	
	**Steroid avoidance + ATG**	**Steroid maintenance + IL2**
1	0.982	0.990
2	0.973	0.962
3	0.964	0.942
4	0.955	0.932
5	0.910	0.913
6	0.883	0.893
7	0.813	0.893

Supplementary Table S4. Risk table to Rejection-free survival up to 7 years according to study arm

Time (years)	Survival Probability	
	**Steroid avoidance + ATG**	**Steroid maintenance + IL2**
1	0.900	0.923
2	0.873	0.894
3	0.873	0.894
4	0.873	0.884
5	0.873	0.873
6	0.863	0.863
7	0.849	0.840
